# Amplicon-sequencing of raw milk microbiota: impact of DNA extraction and library-PCR

**DOI:** 10.1007/s00253-021-11353-4

**Published:** 2021-06-01

**Authors:** Annemarie Siebert, Katharina Hofmann, Lena Staib, Etienne V. Doll, Siegfried Scherer, Mareike Wenning

**Affiliations:** 1 Chair of Microbial Ecology, TUM School of Life Sciences, Technische Universität München, Weihenstephaner Berg 3, 85354 Freising, Germany; 2 Bavarian Health and Food Safety Authority, Veterinärstraße 2, 85764 Oberschleissheim, Germany

**Keywords:** PCR bias, Eukaryotic DNA depletion, Enzymatic lysis, Bead-beating

## Abstract

**Abstract:**

The highly complex raw milk matrix challenges the sample preparation for amplicon-sequencing due to low bacterial counts and high amounts of eukaryotic DNA originating from the cow. In this study, we optimized the extraction of bacterial DNA from raw milk for microbiome analysis and evaluated the impact of cycle numbers in the library-PCR. The selective lysis of eukaryotic cells by proteinase K and digestion of released DNA before bacterial lysis resulted in a high reduction of mostly eukaryotic DNA and increased the proportion of bacterial DNA. Comparative microbiome analysis showed that a combined enzymatic and mechanical lysis procedure using the DNeasy^®^ PowerFood^®^ Microbial Kit with a modified protocol was best suitable to achieve high DNA quantities after library-PCR and broad coverage of detected bacterial biodiversity. Increasing cycle numbers during library-PCR systematically altered results for species and beta-diversity with a tendency to overrepresentation or underrepresentation of particular taxa. To limit PCR bias, high cycle numbers should thus be avoided. An optimized DNA extraction yielding sufficient bacterial DNA and enabling higher PCR efficiency is fundamental for successful library preparation. We suggest that a protocol using ethylenediaminetetraacetic acid (EDTA) to resolve casein micelles, selective lysis of somatic cells, extraction of bacterial DNA with a combination of mechanical and enzymatic lysis, and restriction of PCR cycles for analysis of raw milk microbiomes is optimal even for samples with low bacterial numbers.

**Key points:**

• *Sample preparation for high-throughput 16S rRNA gene sequencing of raw milk microbiota.*

• *Reduction of eukaryotic DNA by enzymatic digestion.*

• *Shift of detected microbiome caused by high cycle numbers in library-PCR.*

**Supplementary Information:**

The online version contains supplementary material available at 10.1007/s00253-021-11353-4.

## Introduction

Amplicon-based high-throughput sequencing undoubtedly facilitates the in-depth characterization of individual complex raw milk (RM) microbiota. It has been applied in recent years to unravel the impact of weather conditions (Li et al. 2018), the health status of the dairy cow (Lima et al. 2018), farm environment, and the management and milking practices (Doyle et al. 2017; Fretin et al. 2018; Metzger et al. 2018; Dahlberg et al. 2020) on the bacterial community composition.

Although the next-generation sequencing technology (NGS) enabled the rise of amplicon-based microbiome analysis, milk protein micelles, and high-fat content (Quigley et al. 2013) of raw milk hamper bacterial DNA extraction. Moreover, in European fresh raw cow’s milk, the aerobic, mesophilic cell count is restricted to a maximum of 5 log colony forming unit (cfu) mL^-1^ (Regulation (EC) No 853/2004), but densities usually range from 3 to 5 log cfu mL^-1^ (Fricker et al. 2011; Mallet et al. 2012; von Neubeck et al. 2015; Fretin et al. 2018; Skeie et al. 2019). The somatic cell count originating from the cow’s immune cells should not exceed 400,000 cells mL^-1^ (Regulation (EC) No 853/2004). Indeed, the diploid bovine genome size of approximately 6000 Mbp (Zimin et al. 2009) is much larger than an average bacterial genome size of 2.5 to 5.8 Mbp, which was found, for instance, in the human gut (Nayfach and Pollard 2015). In addition to the sample matrix properties, the disadvantageous ratio between bacterial and eukaryotic DNA further poses an enormous challenge to bacterial DNA isolation.

Investigations of DNA extraction methods to reduce artifacts have been discussed in the literature for different types of matrices such as feces (Panek et al. 2018), dairy cow rumen (Vaidya et al. 2018), or human breast milk (Douglas et al. 2020) samples. The effective lysis of Gram-negative and Gram-positive bacteria is needed to ensure accurate identification of the relative abundances of biodiversity detected after sequencing (Breitenwieser et al. 2020). With particular emphasis on hard to lyse Gram-positive bacterial cell walls, disruption can include chemical, enzymatic, and mechanical lysis or a combination of several methods.

Amplicon sequencing is a widely employed NGS method to uncover the total taxonomic diversity (Salipante et al. 2014; Sinclair et al. 2015; D’Amore et al. 2016; Cao et al. 2017). The specific target region of the ribosomal 16S rRNA gene sequence amplified during library preparation varies among different studies. However, the variable regions V3-V4 have been commonly used for cow’s milk (Doyle et al. 2017; Cremonesi et al. 2018; Fretin et al. 2018; Li et al. 2018) as well as for human breast milk (Biagi et al. 2017; Murphy et al. 2017) microbiome analysis. In contrast, shotgun metagenomic sequencing relies on whole-genome analysis using a PCR-free sample preparation. One of its advantages over amplicon sequencing is the identification down to the species level instead of operational taxonomic unit (OTU) level. While this method is used more frequently for environmental samples with higher bacterial counts and usually in addition to amplicon sequencing (Vogtmann et al. 2016; Vangay et al. 2018), amplification of target DNA during library preparation is advantageous for raw milk samples due to limited availability of bacterial DNA. Nevertheless, the methodology-driven introduction of bias arises not only from the mode of bacterial lysis for DNA extraction but also from amplification rates during amplicon generation by PCR (Aird et al. 2011; Gohl et al. 2016; Sze and Schloss 2019).

This study investigates the effect of different bacterial lysis methods of commercially available extraction kits on DNA yield as well as the resulting amplicon sequencing data to assess the sample preparation for raw milk microbiome analyses. EDTA- pretreatment and selective lysis (SL) of somatic cells before DNA extraction were tested to increase bacterial DNA yield. Moreover, we addressed the question of whether the cycle number in the library-PCR affects biodiversity as well as species abundances.

## Material and methods

### Raw milk samples

Raw milk samples were collected either directly from a bulk tank of a farm or storage tanks of a dairy. The milk was kept in sterile glass or plastic bottles and was treated without adding preservatives. The samples were refrigerated at 4 °C and processed immediately or latest after 72 h of transportation and storage.

### Bacterial counts

The total mesophilic, aerobic bacterial count of the raw milk was determined by applying the spread-plate method. One-fourth Ringer’s solution (Merck, Darmstadt, Germany) was used for serial dilutions, which were plated on tryptic soy agar (TSA; Oxoid, Basingstoke, Hampshire). Every dilution was plated in duplicate and incubated at 30 °C for 5 days.

### Separation of bacterial cells from raw milk

For optimizing the separation of bacterial cells, two different variants were tested. A volume of 45 mL raw milk was used for initial centrifugation at 13,000×*g* for 20 min at 4 °C. The milk protein content was reduced by adding the chelating agent EDTA to dissociate casein micelles to improve the yield of bacterial DNA extracted from raw milk (Murphy et al. 2002). In each of the two approaches tested, the milk fat fraction was removed, and the skim milk supernatant of the constantly cooled sample was carefully reduced to approximately 1.0 mL. The pellet was resuspended in the remaining skim milk. In the first variant, 3.0 mL of 0.5 M EDTA (Roth, Karlsruhe, Germany) pH 8.0 and 2.0 mL Tris(hydroxymethyl)aminomethane-EDTA (TE)-buffer (10 mM Tris-HCl + 1.0 mM EDTA, pH 7.6) were added to 45 mL of raw milk before centrifugation, gently mixed, and then centrifuged. The second approach was carried out according to Murphy et al. (2002): after the initial centrifugation without EDTA, 300 μL 0.5 M EDTA and 200 μL TE-buffer were added to the resuspended pellet and incubated for 1 min at room temperature (RT) to dissolve the pelleted casein. With both EDTA treatments, the resulting suspension was transferred to a 2.0 mL tube and centrifuged at 16,000×*g* for 1 min at RT. The supernatant was reduced to about 100 μL for subsequent reduction of the eukaryotic DNA. A comparison of both analyses was carried out using four raw milk samples analyzed with three to six replicates each (Supplementary Fig. S1A). Real-time PCR of each replicate was performed in duplicates. For testing different DNA extraction kits and library-PCR cycle numbers, the initial volume of raw milk was increased to 47 mL centrifuged with adding 3.0 mL 0.5 M EDTA.

### Selective reduction of eukaryotic DNA

Selective lysis of eukaryotic cells that originate from the cow was performed to reduce the amount of eukaryotic DNA. Unless otherwise specified, selective lysis was applied to each DNA extraction. One-fourth Ringer’s solution (880 μL when applying SL and 100 μL for treatments without SL) was added to the remaining supernatant (~100 μL) after EDTA pretreatment to resuspend the pellet. Samples without SL were stored at −20 °C until DNA extraction. The suspensions subjected to SL were then treated with 20 μL (20 mg mL^−1^) of proteinase K solution (AppliChem, Darmstadt, Germany) for 30 min at 55 °C and 350 rpm to destabilize the cell membrane of somatic cells (Murphy et al. 2002). Centrifugation was performed at 5.000×*g* for 5 min at RT. Released eukaryotic DNA was removed with the supernatant and the pellet was resuspended in 80 μL PCR-grade water (Sigma-Aldrich, St. Louis, USA). Digestion of residual accessible eukaryotic DNA was performed by adding 10 μL of 10X reaction buffer with MgCl_2_ and 10 μL DNase I (1 U/μL) (ThermoFisher, Waltham, USA) followed by incubation for 20 min at 37 °C and 350 rpm. Enzymes were subsequently inactivated at 85 °C for 10 min and samples were stored at −20 °C until DNA extraction. The impact of SL was tested on each treatment (EDTA-treated RM and pellet) in triplicates, all from the same raw milk sample, and real-time PCR was carried out with two technical replicates (Supplementary Fig. S1A).

### DNA extraction using the DNeasy® PowerFood® Microbial Kit

The DNeasy^®^ PowerFood^®^ Microbial Kit (Qiagen, Hilden, Germany) relies on mechanical lysis by microbeads and column extraction. There are no enzymes mentioned to be contained in the supplied lysis buffer, as the lytic reagent a detergent is given. The protocol was therefore adapted based on modifications by Quigley et al. (2012) and was used for all extractions throughout the optimization process of the sample preparation and library-PCR cycle number experiments (Supplementary Fig. S1A, C): 1.0 μL (25 μg mL^−1^) lysozyme (Roth, Karlsruhe, Germany) and 100 U mutanolysin (Sigma-Aldrich, St. Louis, USA) were added together with 450 μL of the kit’s corresponding MBL buffer to the bacterial suspension and incubated for 60 min at 37 °C and 350 rpm. This was followed by the addition of 10 μL (12.5 mg mL^−1^) proteinase K solution and incubation of 60 min at 55 °C and 350 rpm. Samples were then heated for 10 min at 70 °C, and the lysis suspension was transferred to the kit’s PowerBead tube. The vortexing step (10 min at maximum speed) of the manufacturer’s protocol was replaced by shaking the samples 4×6.5 m/s for 30 s using a FastPrep-24™ instrument. Extraction was further carried out according to the manufacturer’s instructions. To maximize the amount of extracted DNA, the elution volume was reduced to 50 μL, and for the kit comparisons, a final volume of 35 μL PCR-grade water was used. DNA was extracted after 1 min of incubation at RT and 1 min centrifugation (13,000×*g*).

### Comparison of bacterial lysis using different extraction methods

In the optimization process, two modifications of the extraction method using the PowerFood kit were tested. As one alternative variant, the incubation time for the enzymes was reduced to 30 min. The other modification did not include enzymatic lysis but consisted of 6×6.5 m/s for 30 s (bead-beating only, PFwoEL) in a FastPrep-24™ instrument with 1 min cooling on ice after half the time. In addition to the PowerFood kit variants, the extraction of bacterial DNA was evaluated by testing two other commercially available DNA extraction kits, as well as the extraction reagents contained in the PathoProof™ Complete-16 Kit (Thermo Scientific, Waltham, USA) (Table 1 and Supplementary Fig. S1B). While the PathoProof™ Complete-16 Kit and the foodproof^®^ Sample preparation Kit II (Biotecon Diagnostics, Potsdam, Germany) represent enzymatic lysis with a column-based purification, foodproof^®^ StarPrep Two Kit (Biotecon Diagnostics, Potsdam, Germany) relies on bead-beating without further purification. For those three kits, bacterial DNA was extracted according to the manufacturer’s specifications, but starting directly with the lysis solution’s addition and with a final elution of the DNA in a volume of 35 μL. The bead-beating step of the StarPrep Two kit is not precisely specified (cell disruption unit for 8 min at maximum speed) and was modified by performing four consecutive steps at 6.5 m/s for 30 s using a FastPrep 24™ instrument (MP Biomedicals, Santa Ana, USA). DNA extraction using the StarPrep Two kit without column-based purification resulted in a final volume of approximately 70 μL of initially added lysis buffer. For comparison, this volume was reduced to about 35 μL by evaporation. Blank negative controls were used to exclude contaminations during the extraction process.

**Table 1:** DNA extraction kits used and the respective isolation principle

Extraction kit	Lysis method	Principle
PathoProof™ Complete-16 Kit (ThermoFisher)	Enzymes	Buffers (supplied) Lysozyme and proteinase K (supplied) Spin column
foodproof^®^ Sample Preparation Kit II (Biotecon Diagnostics)	Enzymes	Buffers (supplied) Lysozyme and proteinase K (supplied) Glass fiber spin column
foodproof^®^ StarPrep Two Kit (Biotecon Diagnostics)	Bead-beating	Buffer (supplied) Bead-beating No column
DNeasy^®^ PowerFood^®^ Microbial Kit (Qiagen)	Enzymes + bead-beating	Buffers (supplied) Bead-beating Silica membrane spin column Additional steps: Modified according to Quigley et al. (2012): Additional enzymatic lysis (lysozyme, mutanolysin, and proteinase K) Heating at 70 °C for 10 min

### Quantification of bacterial DNA

For assessing different pretreatment methods, the yield of isolated bacterial DNA was measured by real-time PCR using universal 16S rDNA primers. With a final volume of 20 μL, the PCR mixture consisted of 10 μL SYBR® Green Supermix (Biorad, Hercules, USA), 1 μL of 515F primer (GTGCCAGCMGCGCGGTAA), 1.0 μL 806R primer (GGACTACHVGGGTWTCTAAT), both with a concentration of 10 pmol μL^−1^, and 5.0 μL extracted DNA. The following PCR program was used: initial denaturation at 98 °C for 5 min, denaturation at 98 °C for 20 s, annealing at 52.5 °C for 30 s, and final elongation at 72 °C for 40 s using the PCR cycler CFX96 (Biorad, Hercules, USA). Ct values of real-time PCR were calculated by the CFX Maestro™ software (Biorad, Hercules, USA). Bacterial DNA extracted from pure cultures of *Acinetobacter*, *Corynebacterium*, *Kocuria*, *Lactococcus*, *Microbacterium*, *Pseudomonas*, and *Staphylococcus* was used as a reference. A standard consisting of eukaryotic DNA (extracted from raw milk or pure bovine eukaryotic DNA) and different proportions (10%, 1%, and 0.1%) of bacterial DNA were used to quantify the percentage of bacterial DNA in the eluate after DNA extraction.

### Library-PCR for amplicon-sequencing

Based on a two-step approach (Berry et al. 2011), the V3–V4 region of the bacterial 16S rRNA gene was amplified for sequencing library preparation. Since the total DNA extracted from raw milk is a mixture of eukaryotic DNA originating from the cow’s somatic cells and bacterial DNA, concentrations were not adjusted. The two-step PCR was performed in duplicates, triplicates, or quadruplicates (depending on the raw milk sample and cycle number used) using the primers 341F and 785R (Klindworth et al. 2013) and 7.0 μL of DNA extract in the first-step PCR. The final volume of each PCR sample was 20 μL. A modified PCR program was used for amplification, which consisted of initial denaturation at 98 °C for 2 min, 20 cycles (unless otherwise stated) of denaturation at 98 °C for 20 s, annealing at 55 °C for 40 s, elongation at 72 °C for 40 s, and a final extension at 72 °C for 2 min. In the second step, the unique barcode combination and Illumina adaptors were incorporated to obtain the final amplicon. Five microliters of each PCR product of step 1 was used as a template for step 2 of the library-PCR. The same PCR protocol was applied, except for an initial denaturation at 98 °C for 40 s and 10 instead of 20 PCR cycles. Parallel PCRs with the same barcode were pooled and purified by mixing the PCR product with Agencourt AMPure XP beads (Beckman Coulter, Inc., Brea, USA) using an amount of 1.8X the volume of the pooled PCR product. The DNA concentrations were measured by the Qubit™ fluorometer 2.0 with the corresponding dsDNA HS Assay Kit (ThermoFisher, Waltham, USA) and adjusted to 0.5 nM. Samples were sequenced in paired-end mode (2 × 275 nt) on an Illumina MiSeq platform (Illumina Inc., San Diego, CA) using MiSeq Reagent v3 Kits following the manufacturer’s instructions.

### Analysis of sequencing data

The raw 16S rRNA gene amplicon dataset was processed using the web platform Integrated Microbial Next Generation Sequencing (IMNGS) (Lagkouvardos et al. 2016), in which a modified version of UPARSE (Edgar 2013) is implemented. Reads were demultiplexed, forward and reverse read merged, and 10 nucleotides trimmed at each end. The clustering of quality-filtered reads was performed by USEARCH 11.0 (Edgar 2010) at 97% sequence identity. OTUs were screened for chimeric sequences against the Ribosomal Database Project (RDP) database (Cole et al. 2014) using UCHIME (Edgar et al. 2011). SortmeRNA version 4.2 (Kopylova et al. 2012) was used to remove non-prokaryotic OTUs. Taxonomy was assigned by SINA version 1.6.1 (Pruesse et al. 2012) using Silva release 128 as reference database (Quast et al. 2013). Filtered reads were mapped and OTUs occurring at less than 0.25% relative abundance in all samples were discarded to reduce artifacts and spurious OTUs. As this will also remove real but low abundance OTUs, when only few samples are processed and overall richness is low, the dataset was extended by adding raw sequence reads from 100 samples of another project (unpublished data) before demultiplexing and processing using IMNGS. This was particularly necessary for analyzing the impact of cycle numbers in library-PCR. Before data normalization, all samples not part of this study were removed and samples having a minimal read count of 7348 were included in the analysis.

Processed data were analyzed based on the Rhea R-scripts (Lagkouvardos et al. 2017) to perform diversity analyses. Species richness and the Shannon.Effective were calculated to investigate alpha-diversity of the microbiota. Analysis of beta-diversity was conducted to compare microbiota compositions across samples. It was based on the generalized UniFrac distances (Chen et al. 2012) considering the shared microbial composition across samples as well as the phylogenetic distances between OTUs. Visualization of the obtained distance matrices was performed by non-metric Multi-Dimensional Scaling (NMDS).

### Statistical analyses

Statistical analyses were conducted using R version 4.0.2 (R Core Team 2020). For all tests, data normality and variance homogeneity were checked with Shapiro-Wilk and *F* tests. The impact of raw milk treatments prior to bacterial DNA extraction, amplicon concentration, and cycle number was determined using the paired *t*-test, the Welsh *t*-test, or the Wilcoxon Rank Sum test. Differences were considered significant (*p*<0.05) or highly significant (*p*<0.01).

## Results

The sample and library preparation protocol for analysis of raw milk microbiota was optimized to increase bacterial DNA yields and reduce artifacts. The study focused on three steps: (i) reduction of eukaryotic DNA from the cow’s somatic cells by removal of casein and subsequent selective lysis; (ii) bacterial lysis and DNA extraction by either enzymatic lysis, mechanical treatment, or a combination of both to enhance lysis in particular of Gram-positive cells; and (iii) the number of cycles in the library-PCR to check for PCR bias.

### Reduction of eukaryotic DNA by removal of casein and selective lysis of somatic cells

As the cow’s genome is approximately 1000-fold larger than a bacterial genome, there is a large excess of eukaryotic over bacterial DNA. This may impair the proper amplification of 16S rDNA amplicons. Therefore, two pretreatments of milk samples before bacterial DNA extraction aimed at improving DNA yield and PCR efficiency. The main goal was to reduce eukaryotic DNA by selective lysis of somatic cells using proteinase K and digestion of released DNA by DNase I. To exclude a potential loss of target bacterial DNA during selective lysis, the impact of proteinase K on prokaryotic cells was investigated first by inoculating tryptic soy broth (TSB) with pure bacterial cultures of seven Gram-negative and seven Gram-positive strains. Applying SL did neither reduce the amount of DNA extracted compared to the control without SL (relative amount 112% for Gram-negative and 106% for Gram-positive strains) nor did it affect Ct values in quantification by 16S rDNA real-time PCR (data not shown).

**Fig. 1:**
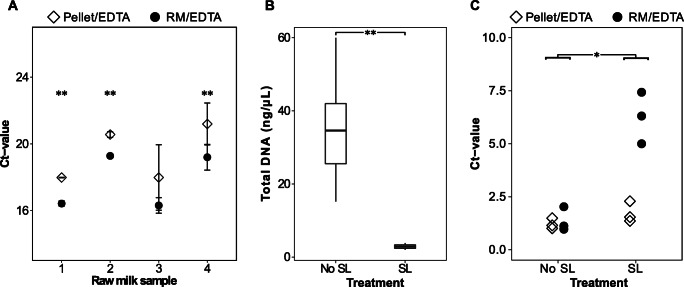
Effect of pretreatment methods on bacterial DNA yield after bacterial lysis and DNA extraction using the DNeasy^®^ PowerFood^®^ Microbial Kit. **A** Detection of bacterial DNA by real-time PCR after EDTA treatments. Selective lysis of somatic cells was performed in each case. Raw milk samples (n=4) were treated with 0.5 M EDTA before (RM/EDTA) or after (Pellet/EDTA) initial centrifugation, each with three to six replicates. Impact of selective lysis (SL: proteinase K and DNase I treatment) on total DNA concentrations (**B**) and proportion of bacterial DNA (%) (**C**) in samples treated with 0.5 M EDTA before (RM/EDTA) or after (Pellet/EDTA) initial centrifugation. n=three replicates for each combination of SL and EDTA treatment, RM: raw milk, EDTA: ethylenediaminetetraacetic acid (*p<0.05, **p<0.01)

For the application of SL using raw milk, the amount of residual casein needs to be diminished in advance by dissolving the casein micelles to ensure the high efficiency of the enzymatic treatment during SL. Here, the addition of EDTA to raw milk before initial centrifugation was compared to EDTA treatment of the pellet obtained after centrifugation. The two different EDTA treatments were carried out with subsequent selective lysis prior to DNA extraction. Bacterial cell counts of the four individual raw milk samples tested ranged from 4.1-5.4 log cfu mL^−1^. When EDTA was used to clarify the opaque, milky pellet, average Ct values reflecting detection of bacterial DNA were between 18 and 21, whereas in the EDTA treated raw milk detection occurred earlier at average Ct values ranging from 16 to 19 (Fig. 1A). For the raw milk samples analyzed, Ct values were reproducibly and, in three cases, highly significantly reduced by 1.6 to 2.0 when EDTA was added to the raw milk instead of the pellet. While without SL DNA concentrations ranged between 15 and 60 ng/μL, values between 2 and 4 ng/μL after SL indicated a reduced amount of total DNA extracted, on average by a factor of 10 (*p*<0.01) (Fig. 1B), confirming the sensitivity of somatic cells towards proteinase K treatment. Accordingly, the determined proportion of bacterial DNA in the DNA extract increased significantly (*p*<0.05) (Fig. 1C).

### Comparison of bacterial lysis using different DNA extraction methods

Bacterial lysis procedures were assessed after pretreatment of milk samples by the addition of EDTA during centrifugation and selective lysis of somatic cells. Three extraction methods were applied consisting of either bead-beating, enzymatic lysis, or a combination of both to determine the method best suited for the isolation of bacterial DNA from fresh raw milk (Table 1 and Supplementary Fig. S1B). For the combined enzymatic and mechanical approach, enzymatic lysis was tested for 0.5 h and 1.0 h. We assessed the impact of the isolation method on DNA yield after library-PCR using four raw milk samples obtained from a farm or the bulk tank of a dairy (bacterial counts ranging from 4.3 to 5.3 log cfu mL^−1^) for each kit in triplicates or duplicates. Further, we examined how methodology affected the detected biodiversity and relative abundances for one farm and one bulk tank milk sample (RM A and RM B), respectively.

### DNA concentration after library-PCR

Because fresh raw milk most often has a low microbial load <5 log cfu mL^−1^, the performance of the extraction kit is of utmost importance. DNA concentrations obtained after library-PCR varied markedly between the samples and kits (Table 2). The enzyme-mediated extraction by PathoProof resulted in distinctly (*p*=0.052) lower amounts of DNA after library-PCR compared to the combined treatment (PowerFood kit, 1.0 h additional enzymatic lysis). This trend was also observed for the enzymatic method by the Sample Preparation kit (*p*=0.06) and was, in particular, pronounced for both kits in samples A and C having lower bacterial counts. Independent from the initial bacterial densities, results indicated a weak efficiency when only enzymatic lysis was applied. While these two kits obtained DNA concentrations of 3.7 ng mL^−1^ and 0.8 ng mL^−1^ on average for sample A, mechanical lysis in the PowerFood kit without enzymatic treatment resulted in a distinctly higher quantity of 17.8 ng mL^−1^. However, mechanical lysis by the StarPrep Two kit without a column-based purification was less efficient for sample A (4.2 ng mL^−1^) but performed better for samples B, C, and D, having 0.5–1.0 log-units higher bacterial counts.

**Table 2:** DNA yield after two-step library-PCR of raw milk samples A–D extracted using various isolation methods

	Sample	A	B	C	D
Bacterial count (log cfu mL^−1^) of raw milk sample		4.3	5.2	4.8	5.3
Lysis method	Kit	DNA yield ng μL^−1^			
Enzymes	PathoProof™ Complete-16 Kit	3.7±0.3	65.5±17.4	17.2±17.0	0.4±0.1
	foodproof^®^ Sample Preparation Kit II	0.8±0.2	3.4±1.3	2.7±2.1	45.9±24.1
Bead-beating	foodproof^®^ StarPrep Two Kit*	4.2±0.8	61.1±2.2	42.8±0.2	57.1±1.7
	DNeasy^®^ PowerFood^®^ Microbial Kit woEL	17.8±10.1	74.0±4.1	n.d.	n.d.
Enzymes + bead-beating	DNeasy^®^ PowerFood^®^ Microbial Kit Modification: 0.5 h of additional EL	14.3±0.6	68.7±5.7	n.d.	n.d.
	DNeasy^®^ PowerFood^®^ Microbial Kit Modification: 1.0h of additional EL	14.8±3.8	89.6±20.1	74.4±14.9	108±9.9

Standard error was calculated from duplicates or triplicates

*woEL* without additional enzymatic lysis, *EL* additional enzymatic lysis, *n.d.* not determined

*p<0.05: significantly lower DNA concentrations compared to the DNeasy^®^ PowerFood^®^ Microbial Kit 1.0hEL

Thus, the PowerFood kit consistently yielded the highest amplicon concentrations, while the modifications in enzymatic treatment with this kit did not have a remarkable consequence on DNA concentrations after the library-PCR. The data demonstrate that regarding DNA yield after PCR, bead-beating is superior to enzymatic lysis and a column-based purification of DNA is particularly advantageous for samples having lower bacterial counts.

### Biodiversity and relative abundances

Besides obtaining sufficiently high amounts of DNA after library-PCR, it is crucial to avoid the underrepresentation or even the loss of specific taxa due to inefficient bacterial lysis. Therefore, amplicon-based microbiome analyses were performed for milk samples A (4.3 log cfu mL^−1^) and B (5.2 log cfu mL^−1^) to uncover the effect of different lysis protocols on the detected community composition. However, in advance of data analysis, three of five replicates obtained with the Sample Preparation kit (enzymatic lysis) were excluded due to comparatively low read counts (Supplementary Table S1) to avoid artifacts in diversity analysis. Although the DNA concentration was adjusted to 12 pmol μL^−1^ in all samples before sequencing, there seems to be a dependence of read counts on initial DNA concentration after library-PCR (Supplementary Table S1) with RM A displaying lower counts than RM B. The reason for this effect is unknown, but insufficient PCR and artifacts in measuring DNA concentration for low-density samples are likely.

**Fig. 2:**
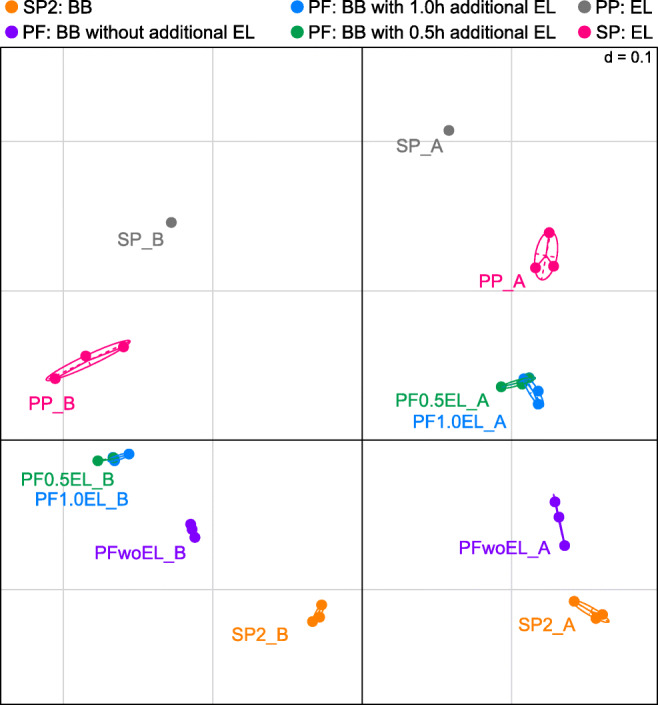
Non-metric MDS plot of generalized UniFrac distances showing the distribution of different extraction protocols of raw milk (RM) A (4.3 log cfu mL^−1^) and B (5.2 log cfu mL^−1^) based on their taxonomic composition. RM was treated either mechanically by bead-beating using foodproof^®^ StarPrep Two Kit (SP2) and DNeasy^®^ PowerFood^®^ Microbial Kit without additional enzymatic lysis (PFwoEL), with enzymatic + mechanical lysis discriminating between 0.5 h and 1.0 h of additional enzymatic lysis (PF0.5EL and PF1.0EL) or with enzymatic based lysis using PathoProof™ DNA extraction (PP) and foodproof^®^ Sample Preparation Kit (SP). BB: bead-beating, EL: enzymatic-lysis. DNA extraction for each kit was performed in duplicates (RM B: SP and PF0.5EL) or triplicates; due to low read counts, only one replicate of SP each was used for data analysis

**Fig. 3:**
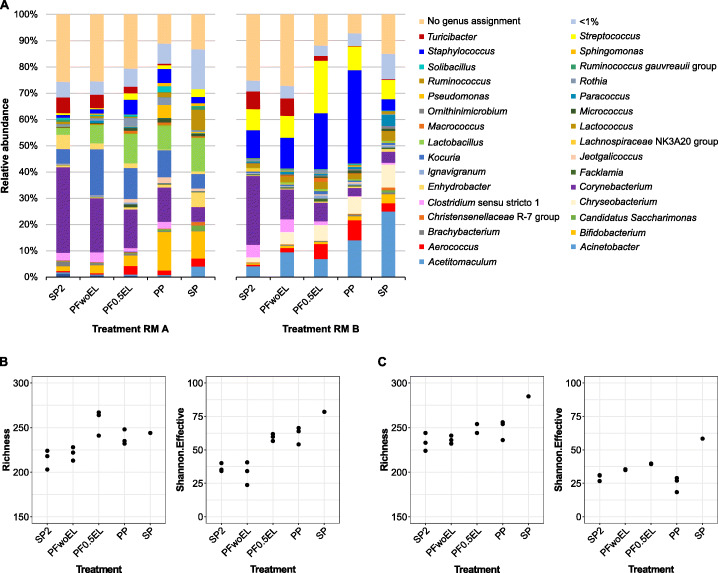
Relative abundance (%) at genus level (**A**) and alpha-diversity indices of farm sample RM A (**B**) and dairy sample RM B (**C**). Data result from two (RM B: SP and PF0.5EL) or three DNA extractions and one replicate of SP used for data analysis. Genera with ≥1% relative abundance are shown in **A**. Bacterial cell count RM A: 4.3 log cfu mL^−1^ and RM B: 5.2 log cfu mL^−1^. RM: raw milk, SP2: foodproof^®^ StarPrep Two Kit, PF: DNeasy^®^ PowerFood^®^ Microbial Kit, woEL: without additional enzymatic lysis, 0.5EL: additional enzymatic lysis for 0.5 h, PP: PathoProof™ Complete-16 Kit, SP: foodproof^®^ Sample Preparation Kit II

For a general comparison of microbiota composition across samples, beta-diversity plots were generated to resolve the contribution of the various extraction methods to the microbial profile (Fig. 2). There is a clear separation of both milk samples A and B (right and left panels), but differences between the extraction protocols appear equally large. Independent of the sample analyzed, there is a shift in beta-diversity from protocols using only bead-beating via samples treated additionally with enzymes (combined approach) to the pure enzymatic treatment (bottom-up). The duration (0.5 h and 1.0 h) of additional enzymatic lysis using the PowerFood kit (PF0.5EL and PF1.0EL) did not affect the microbiota composition. However, beta-diversity without additional enzymatic lysis (PFwoEL) was substantially different and shifted towards the bead-beating-based Star Prep Two kit (SP2). Accordingly, the relative abundance of single genera diverged vastly between different extraction protocols (Fig. 3A and Supplementary Tables S2 and S3). While bead-beating favored the detection of *Corynebacterium*, *Clostridium* sensu stricto 1, and *Turicibacter* (SP2 and PFwoEL), protocols applying enzymatic treatment lead to higher relative abundance of *Bifidobacterium*, *Streptococcus*, and *Staphylococcus* as well as of Gram-negative genera such as *Acinetobacter* and *Chryseobacterium*. Using the DNA extraction reagents of the PathoProof kit being developed to identify mastitis-causing staphylococci is most likely the reason for the comparatively high relative abundance of the genus *Staphylococcus* in RM B (PP).

Alpha-diversity calculations enable the detection of the OTUs’ diversity within a given raw milk sample. We considered the species richness quantifying the number of different OTUs and the Shannon.Effective index adding more weight to the structure of communities and abundances of individual OTUs. While richness values differed only slightly between different extraction variants for RM B (Figs. 3B and 3C), Shannon.Effective was largely increased in both raw milk samples with enzymatic lysis, pointing to a more balanced detection of different taxa.

Eventually, sequencing data underline the relevance of enzymatic lysis for high coverage of biodiversity. However, additional mechanical disruption is pivotal to ensure effective lysis and achieve adequate amounts of DNA after library-PCR. Each technique of cell lysis alone results in either low DNA concentrations and low PCR efficiency (enzymatic lysis) or insufficient coverage of diversity and relative abundances (bead-beating). A combination of both thus seems to be a good compromise.

### Influence of PCR cycle number on diversity estimates

To study whether and to what extent the cycle number in the first step of the two-step library-PCR impacts diversity estimates, we examined the microbial compositions of two farm raw milk and two bulk tank milk samples with bacterial cell counts ranging from 3.7 to 5.3 log cfu mL^−1^. The cycle numbers were varied from 15 to 35 cycles (in increments of 5), and for each variant, two to four replicate PCRs (depending on the raw milk and number of cycles applied; Supplementary Fig. S1C) were pooled for sequencing to obtain sufficient amounts of DNA.

**Fig. 4:**
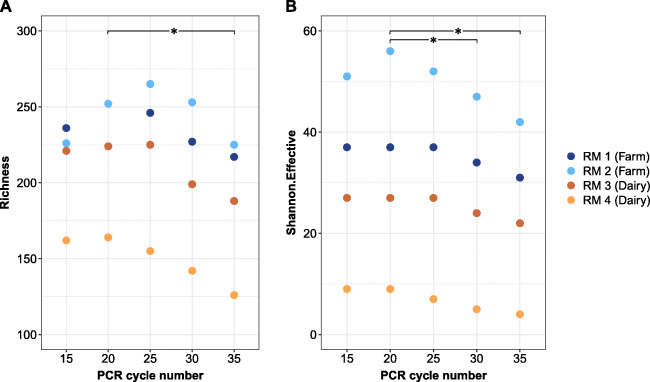
Alpha diversity indices (OTU richness (**A**) and Shannon.Effective (**B**)) for two farm raw milk (RM Farm) and two bulk tank milk (RM Dairy) samples after performing 15, 20, 25, 30, or 35 cycles in the first step of the two-step library-PCR. RM 1: data resulted from duplicates, triplicates, and quadruplicates, depending on the cycle number applied. Bacterial cell count for RM 1: 3.7 log cfu mL^−1^, RM 2: 4.3 log cfu mL^−1^, RM 3: 4.8 log cfu mL^−1^, RM 4: 5.3 log cfu mL^−1^, RM: raw milk (*p<0.05)

The richness in the 15 cycles variant ranged between 162 and 236 OTUs. After a slight increase for most of the samples when the cycle number was shifted to 20 or 25, reduced microbial diversity was detected with increasing cycle numbers, particularly for those with higher bacterial counts (Fig. 4). Thus, with 30 and 35 cycles, a maximum drop in absolute OTU richness values of about 40 was observed for the bulk tank samples 3 and 4 with higher bacterial counts. The shift towards the 35 cycles was even more pronounced for the Shannon.Effective, where the values decreased between 16 and 47% from 20 to 35 cycles. Across all four samples, the effects in comparison to the replicates with 20 cycles were statistically significant (*p*<0.05) for 35 cycles (richness) and 30 and 35 cycles (Shannon.Effective).

**Fig. 5:**
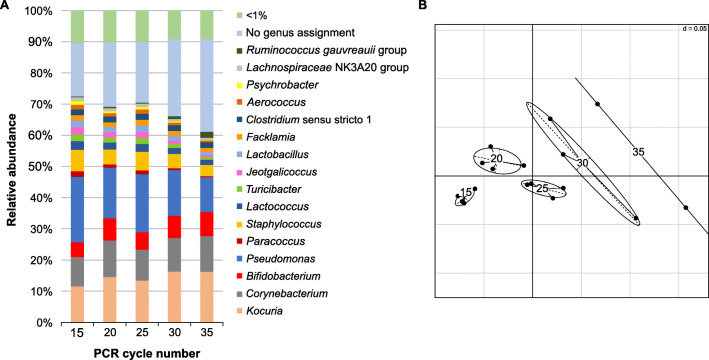
Relative abundance (%) at genus level (**A**) and non-metric MDS plot of generalized UniFrac distances (**B**) showing the taxonomic composition and the related distribution of sample RM 1 (farm, bacterial cell count: 3.7 log cfu mL^−1^). All genera with an abundance of ≥1% are shown in **A**. Library-PCR was performed applying 15, 20, 25, 30, or 35 cycles in the first step of the two-step PCR. Replicate PCRs were barcoded individually. Data result from duplicates, triplicates, and quadruplicates (depending on the cycle number applied). RM: raw milk

Analysis of community composition and beta-diversity revealed that certain genera abundances systematically changed with increasing cycle numbers (Fig. 5 and Supplementary Fig. S2). Results obtained for sample RM 1 (farm) having the lowest cell count (3.7 log cfu mL^−1^) showed the least effects in the alpha-diversity (Fig. 4), but the impact on beta-diversity even between single replicate PCRs barcoded individually was pronounced (Fig. 5B). An increase in relative abundance was detected, e.g., for *Kocuria* (11.5% to 16.2%), *Bifidobacterium* (4.8% to 7.7%), and *Corynebacterium* (9.4%–11.5%), while the relative abundance of *Pseudomonas* (21.1–11.2%) or *Staphylococcus* (6.9%–3.5%) decreased (Fig. 5A). With few exceptions, a trend towards the overrepresentation of more abundant taxa with increasing cycle numbers was observed, whereas genera present at low frequencies were likely to be underestimated or remained undetected. Illustration of single PCR replicates for each cycle number (Fig. 5B) revealed that with more cycles there is not only a shift in relative abundance and diversity but also a reduced reproducibility of single PCRs. As evident from the analyzed shift of the microbial composition, a low cycle number in library-PCR is important to minimize artifacts.

## Discussion

One of the major challenges to be overcome in DNA extraction from raw milk is the low number of bacterial cells, usually <5 log cfu mL^−1^ (Fricker et al. 2011; Mallet et al. 2012; von Neubeck et al. 2015; Fretin et al. 2018; Skeie et al. 2019) combined with the dominance of cow’s eukaryotic DNA. This is not a problem when analyzing, e.g., human fecal samples, as these exhibit bacterial cell counts of approximately 10 to 11 log cfu mL^−1^ or cfu g^−1^ (Whitman et al. 1998; Hopkins et al. 2001), extensively exceeding the amount of human DNA. Sample quantities of <1 g are usually sufficient to extract adequate amounts of DNA for amplicon generation (Claassen et al. 2013; Wagner Mackenzie et al. 2015; Kampmann et al. 2016). The present study aimed at optimizing DNA extraction and library preparation for the analysis of raw cow’s milk microbiota. Besides the unfavorable ratio of the bacterial and somatic cell count, the introduction of artifacts during library-PCR was a further difficulty that had to be overcome.

Concentrating bacterial cells by centrifugation and depleting eukaryotic DNA preceding to specific lysis of bacterial cells were initially chosen to increase the yield of prokaryotic DNA. EDTA chelates calcium ions and leads to the dissociation of casein micelles (Owen and Andrews 1984; Murphy et al. 2002). The direct addition of EDTA to raw milk before initial centrifugation proved to be more efficient than treating the pellet. It prevented the sedimentation of casein micelles into a clotty protein pellet. This not only facilitated the subsequent processing of the pellet but most likely reduced the interference of the casein with proteinase K used in the lysis of somatic cells (Murphy et al. 2002). Significantly lower Ct values obtained in real-time PCR indicated that EDTA added to the raw milk either led to a higher amount of bacterial DNA, an enhanced PCR efficiency, or a combination of both. The application of selective lysis was accompanied by an average of 90% decrease in the total DNA extracted and a simultaneous increase in the percentage of bacterial DNA, which may lead to higher rates of success in PCR. The failure of bacterial DNA amplification and sequencing of raw milk turned out to be problematic in previous studies. Even when applying 35 to 40 cycles in PCR, a total of 18% (Lima et al. 2018) and about 38% (Metzger et al. 2018) of the processed raw milk samples were not successfully amplified in PCR, strongly emphasizing the necessity of increasing the fraction of bacterial DNA.

Sequencing of raw milk and bulk tank milk in our study revealed a dominance of Gram-positive bacteria, supporting the observations previously described for fresh cow’s raw milk (Delbes et al. 2007; Fricker et al. 2011; Lima et al. 2018). Due to the nature of the Gram-positive cell wall, the mode of cell lysis is one of the most crucial steps during DNA extraction. Quigley et al. (2012) previously tested the inclusion of additional steps to the PowerFood kit to isolate DNA from raw milk. They found that the enzymatic treatment by incorporating lysozyme, mutanolysin, and proteinase K with the lysis buffer and a heating step before bead-beating significantly improved DNA yields extracted from milk. In this study, the evaluation of different bacterial lysis methods showed that concerning DNA quantity in library-PCR, bead-beating outperformed enzymatic-based lysis and was significantly more efficient having a column-based purification. Very low amounts of DNA were obtained, in particular for those samples with low bacterial counts. However, the comparative microbial profiling in our study demonstrated the high relevance of enzymatic lysis in addition to bead-beating. Thus, using the combined approach, in particular, *Staphylococcus* and *Streptococcus* were recovered with higher relative abundances than with the bead-beating alone in both analyzed raw milk samples. This is in accordance with findings by Yuan et al. (2012), who observed higher fractions of *Staphylococcus* and *Streptococcus* when using mutanolysin in addition to bead-beating during the extraction of DNA from human-associated species. Comparable observations were reported by Dahlberg et al. (2019), who found an underestimation of two out of three Gram-positive bacteria after using the PowerFood kit without applying additional enzymatic lysis. Additionally, the introduction of methodological bias through insufficient mechanical disruption of hard to lyse Gram-positive taxa belonging to the phyla *Actinobacteria* and *Firmicutes* were described earlier (Biesbroek et al. 2012; Lazarevic et al. 2013; Breitenwieser et al. 2020). To ensure sufficiently high amplicon yields after library-PCR and broad and balanced coverage of the microbial diversity present, the optimization process for bacterial DNA extraction from raw milk resulted in the following protocol: (i) selective lysis after centrifugation of raw milk with EDTA to reduce the fraction of the cow’s eukaryotic DNA and (ii) bacterial lysis using enzymes (lysozyme, mutanolysin, and proteinase K, 0.5 h) followed by bead-beating of 6 × 6.5 m/s using FastPrep-24™.

Apart from the DNA extraction methods, a considerable shift in the biodiversity detected occurred with an increase of cycle numbers in the first step of the two-step library-PCR. Higher cycle numbers (≥30 cycles) contributed to a significant (*p*<0.05) decline of alpha-diversity associated with an alteration of the microbial profile and an impaired reproducibility. PCR amplification affected various bacterial templates differently with increasing cycle numbers. The assumption that PCR favors the amplification of high-frequency bacterial species in a complex community while it causes underrepresentation of less prevalent genera was previously suggested (Gonzalez et al. 2012). Gohl et al. (2016) noticed a decrease in the abundance of *Pseudomonas aeruginosa* with increasing template molecules and PCR cycle numbers, whereas relative abundances of *Bacteroides vulgatus* increased and those of *Escherichia coli* remained relatively constant. Among the genera detected in our samples, we also observed a reduction of relative abundance for the genus *Pseudomonas* of approximately 10% at 35 cycles compared to 15 cycles in one raw milk sample. Hence, a combination of unequal amplification efficiency and different fractions of each taxon represented in the total community may bias the microbial profile (Polz and Cavanaugh 1998; Gonzalez et al. 2012; Gohl et al. 2016), which is most likely considerably reinforced by high cycle numbers in PCR. McGovern et al. (2018) analyzed the V3-V4 region of a low-density mock community consisting of bacteria present in the rumen. They showed that the non-specific background increased with 28 cycles compared to the profile obtained with 20 cycles, which were sufficient to detect the microbiota. In addition to the shift of the biodiversity pattern observed in our raw milk samples, the formation of error and chimeric sequences (e.g., Wang and Wang 1997; Sze and Schloss 2019) can occur with higher cycle numbers.

For raw milk usually having low bacterial counts, the challenge was to find an adequate compromise between generating sufficiently high amplicon amounts, preventing a loss of biodiversity, and reducing individual taxa selection in library-PCR. Finally, one alternative to limit PCR shift would be to restrict the two-step approach to 20 + 10 cycles and pool technical replicates of library-PCR to ensure sufficient DNA levels essential for successful sequencing.

In conclusion, DNA extraction as well as PCR conditions considerably affected the determination of relative template abundances and might, thus, bias the investigation of raw milk microbiomes. Selective lysis of somatic cells and the digestion of eukaryotic DNA led to an enhanced PCR efficiency. Moreover, both an enzymatic and a mechanical lysis step is required, especially for the lysis of Gram-positive bacteria to cover the biodiversity accurately. High cycle numbers in the first PCR step of library-PCR should be avoided to retain as much biodiversity as possible and achieve realistic biodiversity estimates. Although raw cow’s milk is a challenging matrix for the analysis of microbiomes, these adaptations of the sample preparation protocol largely enhance sequencing success and reduce the introduction of bias.

## Supplementary Information

ESM 1 (PDF 661 kb)
