# Supplementary material for: Amplicon-sequencing of raw milk microbiota: impact of DNA extraction and library-PCR
Source: Appl Microbiol Biotechnol. 2021 Jun 1;105(11):4761–73. doi: 10.1007/s00253-021-11353-4 (PMC8195793; doi:10.1007/s00253-021-11353-4)
Supplement: Supplementary file 1 — (PDF 661 kb) [file 253_2021_11353_MOESM1_ESM.pdf]

Journal: Applied Microbiology and Biotechnology

Title: Amplicon-sequencing of raw milk microbiota: Impact of DNA extraction and library-PCR

Authors: Annemarie Siebert[1], Katharina Hofmann[1], Lena Staib[1], Etienne V. Doll[1], Siegfried Scherer,[1] and Mareike Wenning[1,2*]

[1]Chair for Microbial Ecology, TUM School of Life Sciences, Technische Universität München, Weihenstephaner Berg 3, 85354 Freising, Germany

[2]Bavarian Health and Food Safety Authority, Veterinärstraße 2, 85764 Oberschleißheim, Germany
*corresponding author

Correspondence: Mareike Wenning
E-Mail: mareike.wenning@lgl.bayern.de

Supplementary Figures

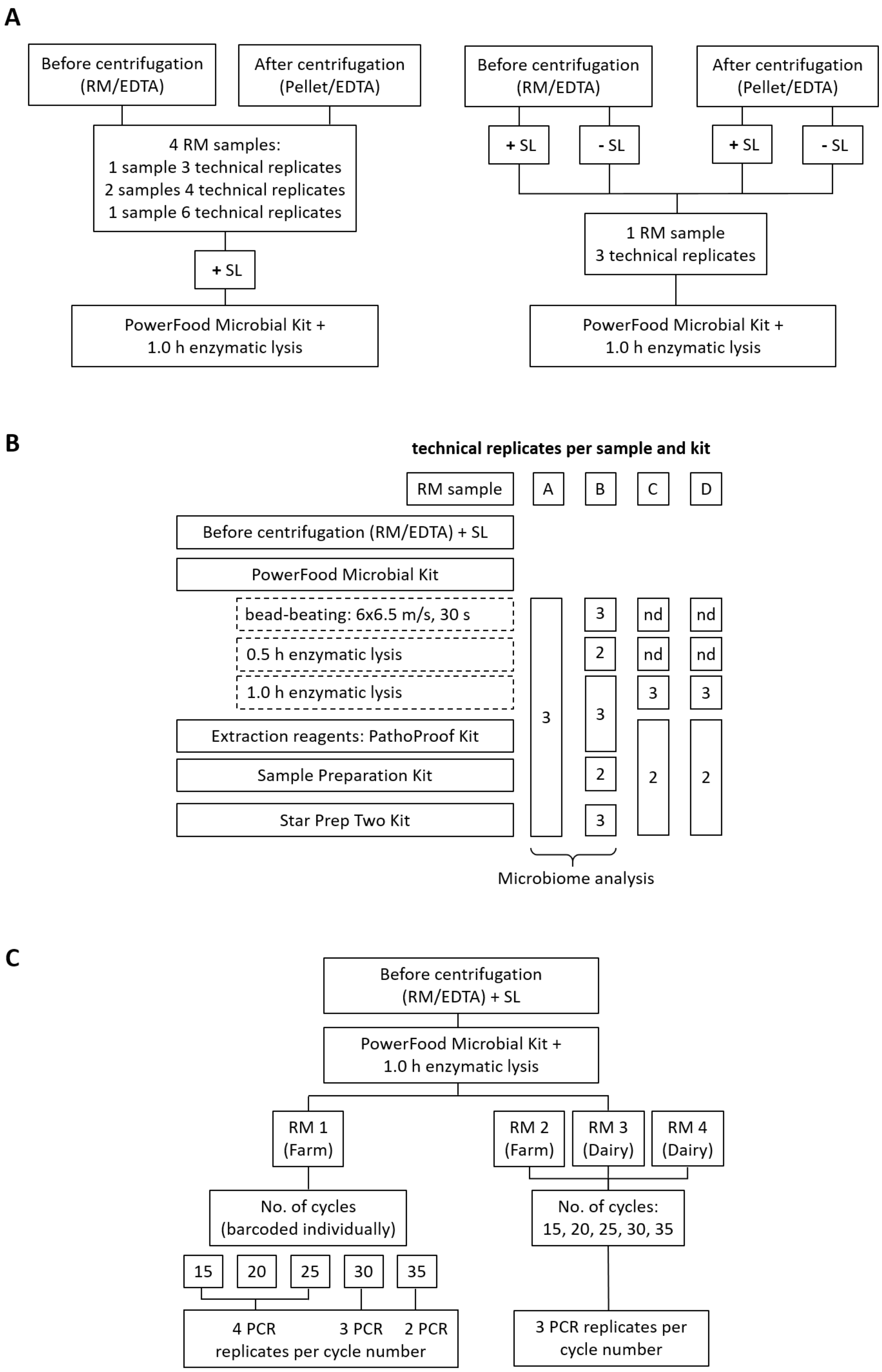

Fig. S1 Schematic overview of the optimization process of bacterial DNA extraction and library-PCR from raw milk (RM) with the respective sample and replicate numbers. Evaluation of (A) pretreatment methods before bacterial lysis, (B) bacterial lysis using different extraction methods, and (C) influence of PCR cycle number in library-PCR. Dashed lines: modifications of the PowerFood kit; EDTA: Ethylenediaminetetraacetic acid; +/- SL: with / without selective lysis; nd: not determined

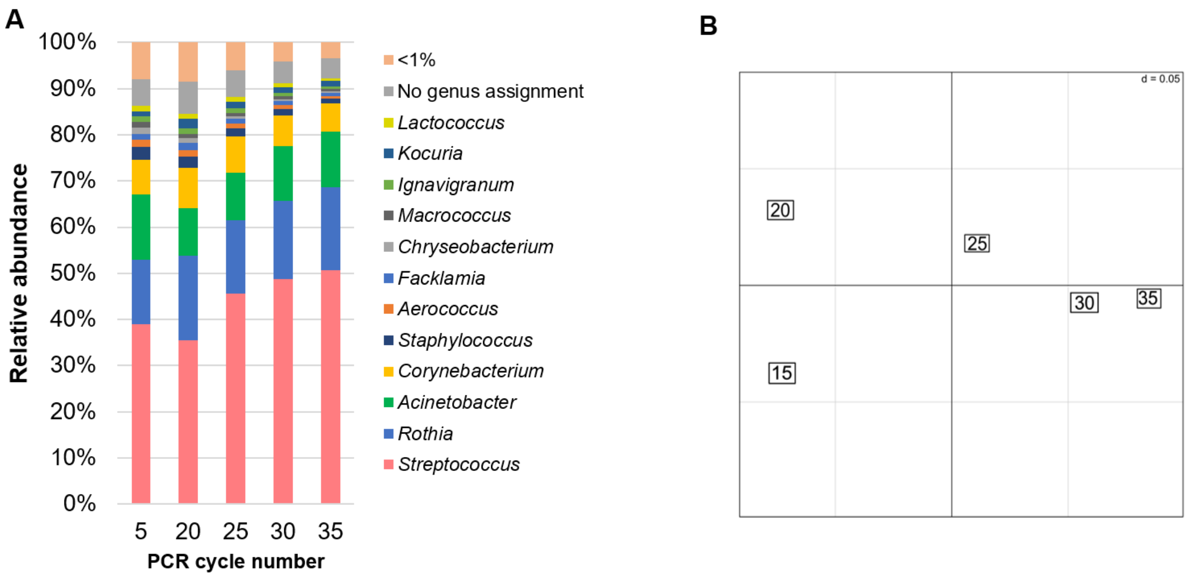

Fig. S2 Relative abundance (%) at genus level (A) and non-metric MDS plot of generalized Unifrac distances (B) showing the composition and the related distribution of sample RM 3 (dairy, bacterial cell count: 4.8 log cfu mL^{-1}). All genera with an abundance of ≥1% are shown in A. Library-PCR was performed applying 15, 20, 25, 30, or 35 cycles in the first step of the two-step library-PCR

Supplementary Tables

Table S1 Number of sequences per sample after data filtering using different kits to extract bacterial DNA from raw milk. OTUs occurring less than 0.25% were discarded. Bacterial cell count of sequenced raw milk A: 4.3 log cfu mL^{-1} and B: 5.2 log cfu mL^{-1}. SP2: foodproof® StarPrep Two Kit; PF: DNeasy PowerFood™ Microbial Kit, woEL: without additional enzymatic lysis, EL: additional enzymatic lysis for 0.5 h or 1.0 h; PP: PathoProof™ Complete-16 Kit; SP: foodproof® Sample Preparation Kit II. DNA extraction and library-PCR were performed in duplicates or triplicates for each kit. Samples removed before normalization are marked in bold

Extraction kit	Raw milk A	Raw milk B
SP2	18,064	56,221
	22,064	158,668
	22,254	296,484
PFwoEL	21,705	61,027
	27,278	238,605
	29,673	352,984
PF0.5EL	25,551	93,555
	26,374	115,055
	32,067	-
PF1.0EL	20,144	107,917
	31,179	139,525
	52,954	243,905
PP	12,552	82,174
	14,600	188,142
	20,523	305,905
SP	**295**	**2,946**
	**1,775**	27,766
	9,780	-

Table S2 Relative abundance (%) of genera in raw milk sample A identified by the amplicon-based sequencing approach after applying different methods for bacterial DNA extraction. Only genera with ≥1% of relative abundance in at least one extraction-method are considered. Data are means from three replicates, SP: two replicates were excluded from data analysis due to low read counts. SP2: foodproof® StarPrep Two Kit; PF: DNeasy PowerFood™ Microbial Kit, woEL: without additional enzymatic lysis, EL: additional enzymatic lysis for 0.5 h or 1.0 h; PP: PathoProof™ Complete-16 Kit; SP: foodproof® Sample Preparation Kit II

	Raw milk A (4.3 log cfu mL^{-1})					
	Extraction method					
	Bead-beating		Enzymes + Bead-beating		Enzymes	
Genera	SP2	PFwoEL	PF0.5EL	PF1.0EL	PP	SP
Acetitomaculum	1.3	0.7	0.5	0.5	0.1	0.2
Acinetobacter	0.6	0.4	0.5	0.4	0.8	3.8
Aerococcus	0.4	0.4	3.2	2.9	1.7	3.0
Bifidobacterium	1.9	3.0	3.9	3.9	14.5	10.3
Brachybacterium	1.6	0.7	0.6	0.6	0.3	0.2
Candidatus Saccharimonas	0.3	0.2	0.3	0.2	0.8	1.9
Christensenellaceae R-7 group	0.3	0.2	0.3	0.3	0.2	1.4
Clostridium sensu stricto 1	2.8	3.6	1.3	1.6	2.6	0.1
Corynebacterium	32.5	20.4	14.5	18.6	12.9	5.5
Enhydrobacter	0.7	0.3	0.8	0.7	1.2	5.6
Facklamia	0.2	0.3	1.2	1.4	0.3	0.2
Jeotgalicoccus	0.2	0.2	1.3	1.6	2.2	1.0
Kocuria	5.6	17.5	11.7	10.4	10.2	5.5
Lachnospiraceae NK3A20 group	5.4	2.2	1.8	1.6	0.3	1.0
Lactobacillus	2.5	6.9	11.0	7.9	9.1	12.7
Micrococcus	0.1	0.3	1.1	1.2	1.6	1.0
Pseudomonas	0.1	0.0	0.1	0.1	4.9	0.4
Rothia	1.0	1.0	3.4	3.2	2.9	0.9
Ruminococcus	0.9	0.7	0.3	0.3	1.7	7.5
Ruminococus gauvreauii group	0.8	0.2	0.3	0.4	0.2	1.2
Solibacillus	0.7	0.4	0.5	0.5	2.2	0.2
Sphingomonas	0.1	0.5	0.2	0.3	1.2	1.0
Staphylococcus	1.1	1.6	5.5	4.3	5.4	2.3
Streptococcus	0.7	0.4	2.4	1.6	1.4	2.9
Turicibacter	5.9	5.1	2.5	2.5	0.6	0.1
<1%	6.0	5.1	6.9	7.4	7.7	15.0
No genus assignment	25.5	25.2	20.3	21.7	11.0	13.2

Table S3 Relative abundance (%) of genera in raw milk sample B identified by the amplicon-based sequencing approach after applying different methods for bacterial DNA extraction. Only genera with ≥1% of relative abundance in at least one extraction method are considered. Data are means from duplicates (PF0.5EL and SP) or triplicates. SP: one replicate was excluded from data analysis due to low read count. SP2: foodproof® StarPrep Two Kit; PF: DNeasy PowerFood™ Microbial Kit, wo EL: without additional enzymatic lysis, EL: additional enzymatic lysis for 0.5 h or 1.0 h; PP: PathoProof™ Complete-16 Kit; SP: foodproof® Sample Preparation Kit II

	Raw milk B (5.2 log cfu mL^{-1})					
	Extraction method					
	Bead-beating		Enzymes + Bead-beating		Enzymes	
Genera	SP2	PFwoEL	PF0.5EL	PF1.0EL	PP	SP
Acinetobacter	3.9	9.4	6.8	6.3	14.0	24.8
Aerococcus	0.6	1.6	5.7	5.3	7.6	3.2
Bifidobacterium	0.6	0.8	0.8	0.9	1.5	3.4
Christensenellaceae R-7 group	0.2	0.1	0.1	0.1	0.2	1.0
Chryseobacterium	1.9	4.7	6.0	5.9	6.4	8.7
Clostridium sensu stricto 1	4.8	4.9	1.5	1.6	0.3	0.7
Corynebacterium	26.2	11.3	7.0	8.2	3.0	4.2
Enhydrobacter	0.3	0.4	0.5	0.4	0.6	1.1
Facklamia	0.4	0.4	1.2	1.1	0.5	0.5
Ignavigranum	0.4	0.4	1.1	1.1	0.5	0.7
Kocuria	0.5	0.6	1.3	1.5	0.5	0.9
Lachnospiraceae NK3A20 group	1.2	0.2	0.1	0.1	0.0	0.1
Lactococcus	1.6	2.2	2.7	2.7	1.9	3.6
Macrococcus	0.1	0.5	1.6	1.4	0.3	0.1
Micrococcus	0.1	0.3	0.5	0.6	1.0	0.9
Ornithinimicrobium	0.2	0.3	0.5	0.5	0.7	1.1
Paracoccus	0.6	0.9	0.5	0.6	0.8	4.2
Rothia	0.8	0.3	1.5	1.6	0.3	0.3
Staphylococcus	10.5	11.7	21.1	20.4	35.4	4.3
Streptococcus	8.1	8.3	19.9	19.1	9.0	7.3
Turicibacter	6.6	6.6	1.8	1.8	0.3	0.3
<1%	4.2	4.8	4.0	12.3	4.8	9.7
No genus assignment	25.1	27.0	11.7	4.3	7.1	14.9
